# Intracellular Environment-Responsive Stabilization of Polymer Vesicles Formed from Head-Tail Type Polycations Composed of a Polyamidoamine Dendron and Poly(l-lysine)

**DOI:** 10.3390/molecules181012168

**Published:** 2013-09-30

**Authors:** Atsushi Harada, Ryota Matsuki, Shin-ichi Ichimura, Eiji Yuba, Kenji Kono

**Affiliations:** Department of Applied Chemistry, Graduate School of Engineering, Osaka Prefecture University, 1-1 Gakuen-cho, Naka-ku, Sakai, Osaka 599-8531, Japan; E-Mails: journey0215@gmail.com (R.M.); ichimura.shinichi@kao.co.jp (S.I.); yuba@chem.osakafu-u.ac.jp (E.Y.); kono@chem.osakafu-u.ac.jp (K.K.)

**Keywords:** nanocapsule, drug delivery system, disulfide bond, glutathione, head-tail type polycation

## Abstract

For the development of effective drug carriers, nanocapsules that respond to micro-environmental changes including a decrease in pH and a reductive environment were prepared by the stabilization of polymer vesicles formed from head-tail type polycations, composed of a polyamidoamine dendron head and a poly(l-lysine) tail (PAMAM dendron-PLL), through the introduction of disulfide bonds between the PLL tails. Disulfide bonds were successfully introduced through the reaction of Lys residues in the PAMAM dendron-PLL polymer vesicles with 2-iminothiolane. The stabilization of PAMAM dendron-PLL polymer vesicles was confirmed by dynamic light scattering measurements. In acid-base titration experiments, nanocapsules cross-linked by disulfide bonds had a buffering effect during the cellular uptake process. The PAMAM dendron-PLL nanocapsules were used to incorporate the fluorescent dyes rhodamine 6G and fluorescein as a drug model. Cationic rhodamine 6G was generally not released from the nanocapsules because of the electrostatic barrier of the PLL membrane. However, the nanocapsules were destabilized at high glutathione concentrations corresponding to intracellular concentrations. Rhodamine 6G was immediately released from the nanocapsules because of destabilization upon the cleavage of disulfide bonds. This release of rhodamine 6G from the nanocapsules was also observed in HeLa cells by laser confocal microscopy.

## 1. Introduction

The self-assembly of macromolecules is often used as an indispensable molecular tool in functional nanomaterial engineering [1,2,3,4,5,6,7]. Block copolymer self-assembly giving spherical or cylindrical micelles and vesicles have especially been used because they combine the characteristic features of synthetic polymers, including solubility and rubber elasticity, with those of polypeptides, including secondary structure, functionality and biocompatibility. Block copolymer-based micelles and vesicles with intracellular environment- (pH, enzyme and reductive environment) responsive behaviors are of special interest for drug delivery system (DDS) carriers [8,9,10,11,12]. For DDS, the drug carriers must dissociate to release entrapped drugs at the target site in response to stimuli in the intracellular environment. For example, polymer micelles containing disulfide cross-linkages within the core have been reported to show effective destabilization in drug delivery. They should be stable in the extracellular environment and promptly release the entrapped drug within the target cell upon the cleavage of disulfide bonds. We have previously investigated head-tail type polycations composed of a polyamidoamine dendron and poly(l-lysine) (PAMAM dendron-PLL) as a carrier in the DDS field [13,14,15,16]. Although PAMAM dendron-PLL was initially designed and synthesized for application as a non-viral gene vector, we recently found that PAMAM dendron-PLL could spontaneously form monodisperse polymer vesicles (self-assembly) through a coil-to-helix transition of PLL tails [17]. The preparation of hollow nanocapsules was carried out by the stabilization of irreversible cross-linkages between Lys residues and PLL tails in polymer vesicles [18].

**Figure 1:**
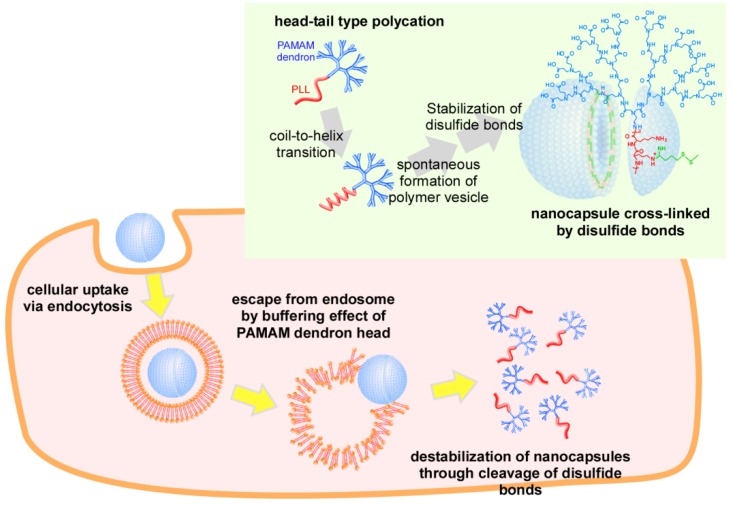
Schematic image of intracellular environment-responsive nanocapsules prepared from head-tail type polycations by the stabilization of disulfide bonds.

In this study, we investigated the stabilization of PAMAM dendron-PLL polymer vesicles by the introduction of disulfide bonds between PLL tails for the application of PAMAM dendron-PLL nanocapsules as a carrier in DDS (Figure 1). The PAMAM dendron head has tertiary amines in the interior, and can contribute to an escape from endosomes or lysosomes in the cellular uptake process because of a buffering effect. Disulfide bonds can also cleave in a reductive environment in cytoplasm, and PAMAM dendron-PLL nanocapsules will be destabilized under these conditions. These properties indicate that PAMAM dendron-PLL nanocapsules that are cross-linked by disulfide bonds can function as carriers in DDS. The stabilization of PAMAM dendron-PLL polymer vesicles has been successful by the introduction of disulfide bonds in a reaction between the Lys residues in a PLL tail and 2-iminothiolane. Nanocapsules have unique properties as drug carriers, and cationic molecules like rhodamine 6G can be effectively incorporated in nanocapsules without being released under physiological conditions. However, nanocapsules can be destabilized under high glutathione concentrations, and rhodamine 6G will be immediately released under these conditions. Furthermore, the release of rhodamine 6G in cytoplasm was confirmed by laser confocal microscopy. These features of PAMAM dendron-PLL nanocapsules cross-linked by disulfide bonds can thus be used for effective drug delivery in the future.

## 2. Results and Discussion

### 2.1. Preparation of Nanocapsules Stabilized by Disulfide Bonds

**Figure 2:**
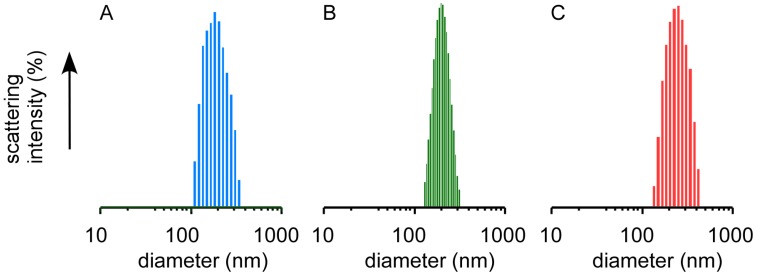
Size distribution of PAMAM dendron-PLL polymer vesicles before (**A**) and after (**B**) reaction with IT in MeOH/H_2_O and nanocapsules (**C**) in H_2_O.

Nanocapsules stabilized by disulfide bonds were prepared by the introduction of SH groups to the Lys residues in the PLL tails of PAMAM dendron-PLL polymer vesicles by a reaction between Lys residues and 2-iminothiolane (IT). Figure 2 shows the size distributions of the PAMAM dendron-PLL polymer vesicles before and after reaction with IT, and PAMAM dendron-PLL nanocapsules after dialysis against distilled water. The average diameter and the polydispersity index of the samples at each preparation step for both the nanocapsules stabilized by IT and ethylene glycol diglycidyl ether (EGDE) as cross-linking reagents are summarized in Table 1. The use of EGDE as a cross-linking reagent provided PAMAM dendron-PLL nanocapsules that were irreversibly stabilized [18]. PAMAM dendron-PLL polymer vesicles were prepared according to the method previously reported [17]. The PLL tail of the PAMAM dendron-PLL had a random coil conformation in a MeOH/water mixture (1:1 by vol.). An increase in MeOH content in the mixed solvent to 80 vol% MeOH induced a coil-to-helix transition in the PLL tails, and the PAMAM dendron-PLL spontaneously formed polymer vesicles with an extremely narrow size distribution. A polydispersity index of less than 0.1 for PAMAM dendron-PLL polymer vesicles indicates a narrow size distribution. Even after the addition of IT to the PAMAM dendron-PLL polymer vesicles, the polymer vesicles maintained their unimodal size distribution. The stabilization of polymer vesicles, *i.e*., the preparation of nanocapsules was confirmed by DLS measurements after dialysis against distilled water. The PAMAM dendron-PLL that was not reacted with the cross-linking reagent was found to be a water-soluble polymer, and PAMAM dendron-PLL polymer vesicles without stabilization broke down into a dispersed state of the PAMAM dendron-PLL by dialysis against water. Dispersed PAMAM dendron-PLL cannot be detected by DLS because of its very low light scattering intensity. However, the PAMAM dendron-PLL polymer vesicles that were reacted with IT were detected by DLS even after dialysis against water and its unimodal size distribution was maintained as shown in Figure 2C. This indicates that the PAMAM dendron-PLL polymer vesicles were stabilized by the disulfide bond between the SH groups that were introduced to the Lys residues by the reaction between IT and Lys residues. Dialysis against water provided an increase in the average diameter. A size change was also observed during the stabilization of PAMAM dendron-PLL polymer vesicles using EGDE as a cross-linking reagent [18]. PAMAM dendron-PLL nanocapsules in the aqueous medium swelled because of the protonation of Lys residues in the PLL tail of the PAMAM dendron-PLL.

**Table 1:** DLS data for PAMAM dendron-PLL polymer vesicles and nanocapsules.

Cross-linker	Average diameter (nm) [polydispersity index]		
	Before cross-linking	After cross-linking	After dialysis
2-iminothiolane (IT)	187 [0.08]	194 [0.06]	218 [0.09]
ethylene glycol diglycidyl ether (EGDE)	187 [0.08]	194 [0.07]	238 [0.08

**Figure 3:**
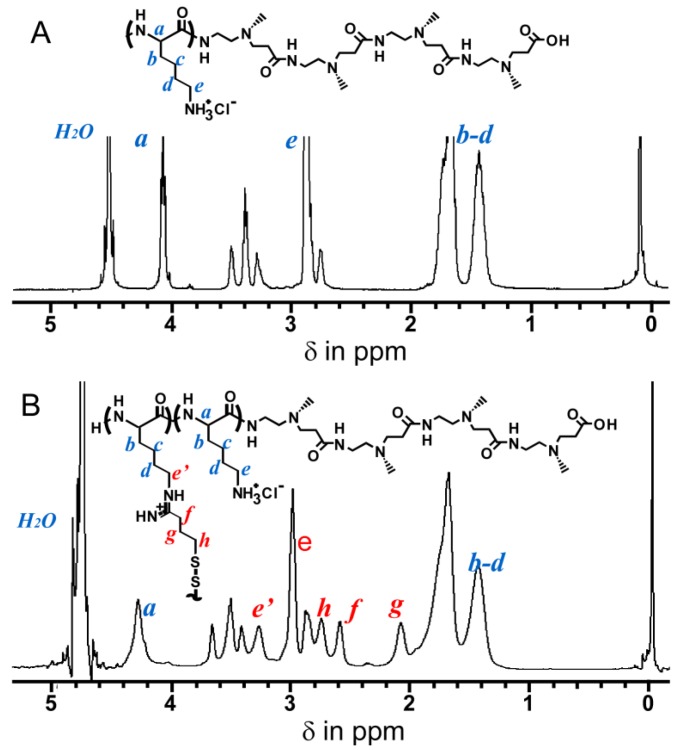
^1^H-NMR spectra of PAMAM dendron-PLL before (**A**) and after (**B**) reaction with IT in D_2_O.

The reaction rate between IT and the Lys residue was determined by ^1^H-NMR. Figure 3 shows the ^1^H-NMR spectra for the PAMAM dendron-PLL before and after reaction with IT. After the reaction with IT new peaks were present at 2.0–2.8 ppm and 3.3 ppm. The IT reaction rate was calculated from the peak area ratio of the CH_2_CH_2_ peak (2.6 ppm) coming from the IT against the α-CH of the Lys residue (4.3 ppm) and the reaction rate of IT to Lys residue was determined to be 22 mol%. This value does not represent the cross-linking density. Considering steric factors, it is difficult to form disulfide bonds with all the introduced SH groups. Some of the introduced SH groups formed disulfide bonds between the PLL tails in the polymer vesicle, and the PAMAM dendron-PLL polymer vesicles were stabilized by disulfide bonds between the PLL tails.

**Figure 4:**
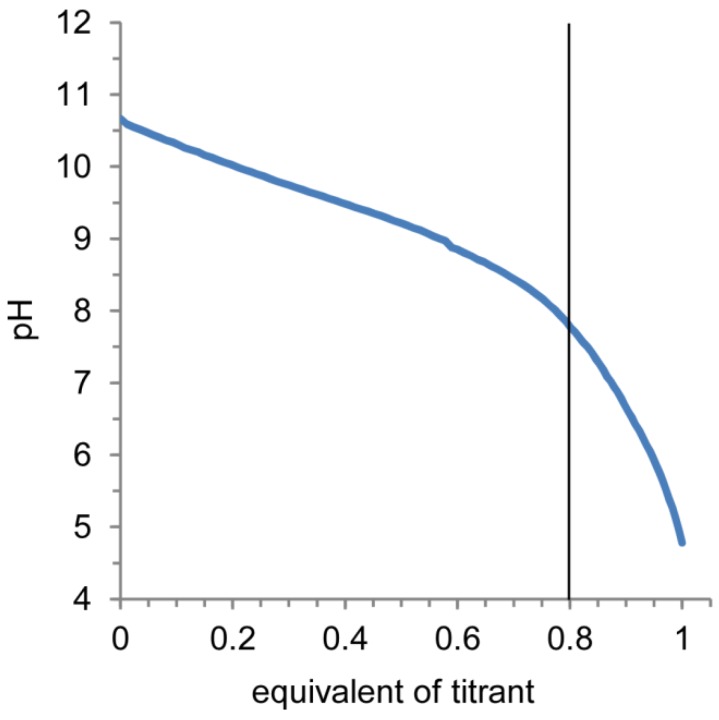
Acid-base titration curve of disulfide-bonded PAMAM dendron-PLL nanocapsules.

An acid-base titration was performed to confirm the response of nanocapsules against pH change. Figure 4 shows the obtained acid-base titration curve (pH *vs*. equivalents of titrant) for the disulfide-bonded PAMAMA dendron-PLL nanocapsules. Two independent deprotonation processes were observed and bound at 0.8 equivalents of titrant. Deprotonation at <0.8 equivalents of titrant was from the deprotonation of tertiary amino groups in the interior of the PAMAM dendron block and the carboxylate groups at the periphery of the PAMAM dendron block. Additionally, deprotonation at >0.8 equivalents of titrant was from the primary amines of the PLL block. Importantly, the addition of titrant was required to decrease the pH from 7.4, indicating that the nanocapsules had a buffering ability during the cellular uptake process via endocytosis.

### 2.2. Release of Model Compounds from Nanocapsules and Cytotoxicity of Nanocapsules

**Figure 5:**
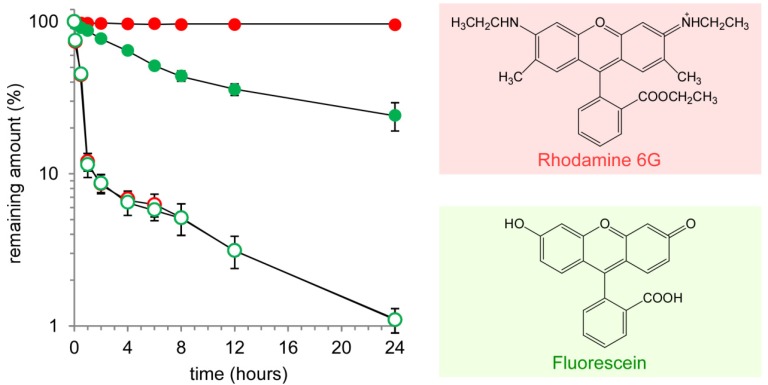
Time course of the remaining amounts of rhodamine 6G (red) and fluorescein (green) in a dialysis cartridge.

The drug containment ability of the nanocapsules was evaluated using rhodamine 6G (Rh6G) and fluorescein (Flu) as model compounds. Figure 5 shows the release profiles for Rh6G and Flu entrapped in the nanocapsules, and free Rh6G and Flu, in which the remaining amounts of Rh6G or Flu in the dialysis cassettes were determined by fluorescence measurements. Free Rh6G and Flu were immediately released (open symbols) and only 10% Rh6G and Flu remained after 1 h of incubation. This demonstrates that the released Rh6G and Flu from the nanocapsules could immediately diffuse to the outer phase. For both the Rh6G and Flu entrapped in the nanocapsules the prolonged release effect was confirmed (closed symbols). Interestingly, there was a remarkable difference in the prolonged release effect between Rh6G and Flu. Flu was gradually released, and 25% remained after 24 h of incubation. On the other hand, more than 95% Rh6G was contained in the nanocapsules. This significant difference might be due to differences in the charge properties of Rh6G and Flu. At pH 7.4, Rh6G has positive charges and Flu has negative charges. As for the nanocapsules, an inner and an outer aqueous phase are separated by a PLL membrane, which is positively charged at pH 7.4. Flu can distribute over the cationic PLL membrane as counter ions and diffuse to the outer aqueous phase because of the difference in concentration between the inner and outer aqueous phase. However, it is more difficult to distribute cationic Rh6G throughout the cationic PLL membrane. As a result, almost no Rh6G was released from the PAMAM dendron-PLL nanocapsules. This release property of disulfide-bonded nanocapsules was also observed for nanocapsules irreversibly cross-linked by covalent bonds using EGDE as a cross-linking reagent.

Dyes in the nanocapsules and the free dyes are represented by closed and open symbols, respectively. The data are presented as the average of three experiments ± SD.

**Figure 6:**
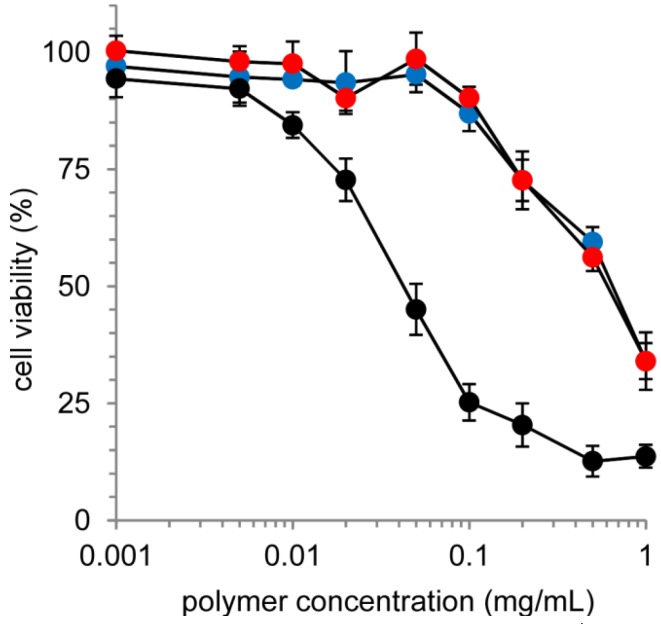
Cell viability of HeLa cells treated by disulfide-bonded (red) and cross-linked (blue) nanocapsules cross-linked by and a non-cross-linked PAMAM dendron-PLL (black).

Although the cationic charge properties of the PLL membrane in the nanocapsules provides a unique drug containment ability, as for the cationic polymer, there are generally concerns about the potential for toxicity. The cytotoxicity of nanocapsules toward cultured cells (HeLa cells) was evaluated by an MTT assay (Figure 6). Although the non-cross-linked polymer (PAMAM dendron-PLL) exhibited a significant cytotoxicity toward HeLa cells, the cross-linking of nanocapsules effectively prevented cytotoxicity. The IC_50_, which is the concentration showing the 50% cell viability, of the PAMAM dendron-PLL polymers and nanocapsules were determined to be 0.042 mg/mL and 0.62 mg/mL, respectively. This significant decrease in cytotoxicity was because of the difference in cellular membrane interactions. The cationic PLL tail of the PAMAM dendron-PLL polymer can interact directly with the cell membrane and thus damage the cellular membrane. On the other hand, the cationic PLL tails do not directly interact with cellular membranes. The hollow architecture of the nanocapsules might provide a remarkable reduction in cytotoxicity.

Cell viability was evaluated by an MTT assay. The cells (5 × 10^4^ per well) were treated with the samples for 4 h, and were then cultured in a medium containing 10% FCS for 20 h. The data are presented as the average of three experiments ± SD.

### 2.3. Response of Nanocapsules to a Reductive Environment

**Figure 7:**
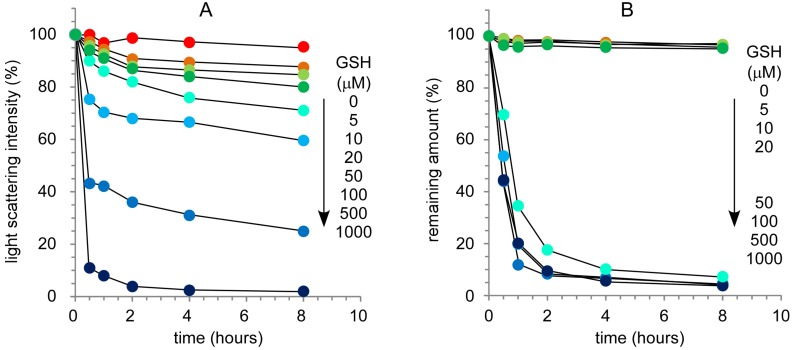
Effect of glutathione concentration on the stability of disulfide-bonded nanocapsules (**A**) and the release of rhodamine 6G from nanocapsules (**B**).

It is well known that the cytoplasm is a reducing environment and that the glutathione (GSH) concentration in the cytoplasm is high (0.5–10 mM) compared with the extracellular environment (~10 µM) [19]. The stability of disulfide-bonded nanocapsules was evaluated from the change in light scattering intensity at varying GSH concentrations, in which the light scattering intensity is very sensitive to a change in the molecular weight of the solute. Figure 7A shows the time course of relative light scattering intensity of nanocpasules at varying GSH concentrations. The light scattering intensity gradually decreased with an increase in incubation time, and the decrease was dependent on the GSH concentration. This indicates that the nanocapsules could be destabilized under reducing environment conditions. This suggests that drug molecules might be released from the nanocapsules through the destabilization of nanocapsules at high GSH concentrations.

The release of Rh6G from nanocapsules at varying GSH concentrations was evaluated by dialysis (Figure 7B). The release of Rh6G did not depend on GSH concentration and two types of release profiles at high and low GSH concentrations were observed. At a low GSH concentration ([GSH] = 0, 5, 10 and 20 µM) the release of Rh6G was not observed. On the other hand, Rh6G was immediately released from nanocapsules at a high GSH concentration ([GSH] = 0.05, 0.1, 0.5 and 1.0 mM). Although the stability of nanocapsules is dependent on GSH concentration as shown in Figure 7A, the difference in the effect of GSH concentration on stability and Rh6G release indicates that a critical condition exists for the collapse of the PLL membranes in nanocapsules. A critical GSH concentration for the release of Rh6G exists and is between 20 and 50 µM. As a result, it was confirmed that disulfide-bonded PAMAM dendron-PLL nanocapsules gave almost no release of Rh6G at an extracellular GSH concentration of ~10 µM, and that the prompt release of Rh6G was induced at intracellular GSH concentrations of 0.5–10 mM.

**Figure 8:**
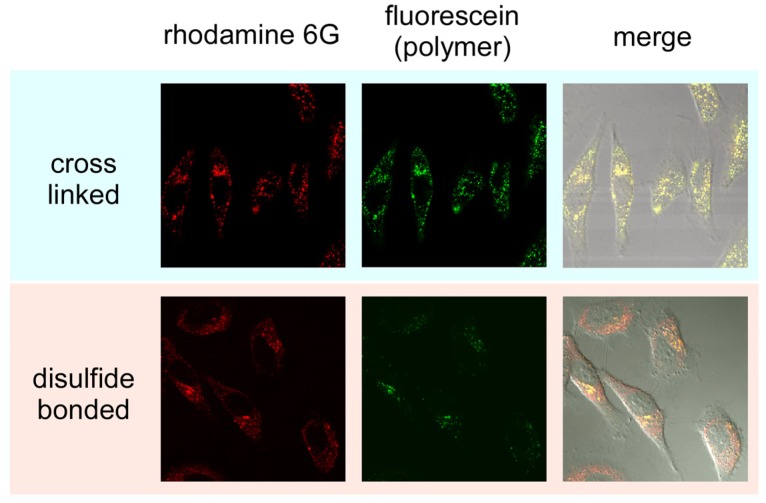
Confocal microscope images of HeLa cells treated by cross-linked and disulfide-bonded nanocapsules.

Finally, the release of Rh6G from nanocapsules in the cultured cells (HeLa cells) was confirmed by laser confocal microscope observations. In this experiment, nanocapsules were covalently labeled with FITC. Figure 8 shows confocal images of HeLa cells treated with disulfide-bonded and cross-linked nanocapsules. In the case of cross-linked nanocapsules both Rh6G (red) and FITC (green) fluorescence dots were observed in the cytoplasm. Additionally, only yellow dots were observed in the confocal laser scanning microscope image overlaid with a differential interference contrast image because of the complete overlap of the distribution of green and red fluorescence dots. This overlap corresponds to an equal distribution of Rh6G and PAMAM dendron-PLL indicating that Rh6G was not released from the cross-linked nanocapsules, even in the cells. On the other hand, for the HeLa cells treated with disulfide-bonded nanocapsules, red fluorescence coming from Rh6G spread in the cytoplasm and the number of yellow dots dramatically decreased in the merged image. This indicates the release of Rh6G from nanocapsules in the cytoplasm through the destabilization of the nanocapsules response in a reductive environment.

## 3. Experimental

### 3.1. Materials

Polyamidoamine dendron-poly(l-lysine) block copolymer (PAMAM dendron-PLL), which had a PAMAM dendron head with a 3.5th generation and a PLL tail with a 93 polymerization degree, was synthesized according to a previous report [13,17,18]. 2-Iminothiolane hydrochloride (IT) was purchased from Sigma-Aldrich (St. Louis, MO, USA). Ethylene glycol diglycidyl ether (EGDE), glutathione reduced form (GSH), rhodamine 6G (Rh6G) and fluorescein (Flu) were purchased from Tokyo Chemical Industry Co. Ltd. (Tokyo, Japan).

### 3.2. Preparation of Nanocapsules Cross-Linked by Disulfide Bonds

Self-assembled PAMAM dendron-PLL polymer vesicles were prepared according to a previous report. Briefly, PAMAM dendron-PLL was dissolved in a 50:50 v/v mixture of distilled water and methanol, and then MeOH was added dropwise with vigorous stirring to give an 80 vol% methanol solution. The formation of PAMAM dendron-PLL polymer vesicles with a narrow size distribution was confirmed by dynamic light scattering (DLS). DLS measurements were carried out at 25 °C using an ELS-8000 (Otsuka Electronics Co., Ltd., Osaka, Japan) equipped with a He/Ne ion laser (λ = 633 nm). The DLS measurements utilized a 90° detection angle: the average diameter was calculated by the Stokes-Einstein equation, and the size distribution was obtained by CONTIN analysis. For the introduction of SH groups to PLL tails, 2-iminothiolane (IT) in H_2_O/MeOH at 80 vol% MeOH were added to a solution of the PAMAM dendron-PLL polymer vesicles, in which the molar ratio of IT and Lys residue was 0.25. After 1 d of reaction, the unreacted IT was removed by dialysis against distilled water and cross-linked PAMAM dendron-PLL polymer vesicles in the form of nanocapsules were obtained. The reaction rate of IT to Lys residue was determined by ^1^H-NMR (JNM-ECX400, JEOL Ltd., Tokyo, Japan).

### 3.3. Dye Release Experiments from Nanocapsules

The fluorescent dyes, Rh6G and Flu, were loaded by mixing with PAMAM dendron-PLL polymer vesicle solution in H_2_O/MeOH at 80 vol% of MeOH. The stabilization of polymer vesicles were performed by same procedures with the nanocapsule preparation as described above. The unloaded dyes were removed by dialysis against distilled water. The release of the fluorescent dyes, Rh6G and Flu, was evaluated by dialysis. Samples of 3.0 mL were put into a dialysis cassette (Slide-A-Lyzer Dialysis Cassette, Thermo Scientific, Rockford, IL, USA) with MWCO 10000, and the dialysis cassettes were immersed in 1.0 L phosphate buffered saline at various concentrations of GSH (0, 5, 10, 100, 500 and 1,000 µM) at 37 °C. 200 µL samples were collected from the dialysis cassette at predetermined times and fluorescence measurements were performed at 25 °C using an FP-6500 spectrofluorimeter (JASCO, Tokyo, Japan). The excitation wavelength and emission range was 527 nm and 530–650 nm for rhodamine 6G and 495 nm and 500–600 nm for fluorescein. The remaining Rh6G and Flu in the dialysis cassettes were calculated using calibration curves for each dye.

### 3.4. *In vitro* Cytotoxicity and Laser Confocal Microscope Observations

The cytotoxicity of the nanocapsules and the PAMAM dendron-PLL was evaluated by MTT assay. HeLa cells were incubated with the samples for 4 h without fetal calf serum (FCS). The culture medium was replaced with 0.2 mL Dulbecco’s modified Eagle medium (DMEM) with 10% FCS containing 40 µL of MTT dissolved in PBS (10 mg/mL) added to each well. After 3 h of incubation the medium was removed and the cells were solubilized in 500 µL of 2-propanol containing 0.1 M HCl. The viable cells were counted from the absorbance at 490 nm using an ARVOSX multilabel counter (Perkin Elmer, Turku, Finland).

To evaluate the intracellular distribution of rhodamine 6G-entrapped nanocapsules, the nanocapsules were labeled using fluorescein isothiocyanate (FITC), in which PAMAM dendron-PLL and FITC were reacted in a 50 mM borate buffer (pH 8.5) and unreacted FITC was removed by dialysis against distilled water. HeLa cells were seeded in 0.5 mL of DMEM supplemented with 10% FCS in glass-bottom dishes at 2 × 10^5^ cells per dish the day before the uptake experiments. The cells were washed with PBS and then covered with DMEM (1 mL). The nanocapsule solutions were gently added to the cells and the solutions were incubated at 37 °C for 24 h. The cells were washed with PBS and confocal laser scanning microscope observations of the cells were performed using a laser scanning microscope (LSM 5 EXCITER, Carl Zeiss Co., Ltd., Oberkochen, Germany).

## 4. Conclusions

Self-assembled polymer vesicles formed from head-tail type polycations, which were composed of a PAMAM dendron head and a PLL tail, were stabilized by the introduction of disulfide bonds through the reaction of Lys residues in PLL tails with 2-iminothiolane. When the cationic dye rhodamine 6G, was incorporated into the PAMAM dendron-PLL nanocapsules, the dye was not released because of the electrostatic barrier in the cationic cross-linked PLL membrane. However, when the glutathione concentration was increased to a concentration corresponding to that found in an intracellular environment the nanocapsules were destabilized through the cleavage of disulfide bonds and the incorporated dyes were immediately released from the nanocapsules. The effective release of a cationic dye that recognizes the intracellular reductive environment was also observed in cultured cells. The obtained results showing intracellular specific release without release in the extracellular environment strongly indicates the applicability of PAMAM dendron-PLL nanocapsules cross-linked by disulfide bonds as a carrier in effective drug delivery.
